# 

**Published:** 1895-11

**Authors:** 


### CONTENTS—NOVEMBER, 1895.—VOL. XI., NO. 5.

ORIGINAL COMMUNICATIONS—

A Case of Simple Melanoma of the Iris, with (associated) Symptoms Simulating Simple Non-Inflammatory Glaucoma. Joseph A. Mullen, M. D., Houston . . . . . 221

Clinical Cases. Robert T. Morris, M. D., and P. H. Cronin, M. D. . 225

Remarks on the Surgical Treatment of Chlolelithiasis. Hugh M. Taylor, M. D., Richmond, Va . . . . . 228

CORRESPONDENCE—

New Uterine Forceps. Thomas J. Turpin, M. D . . . . . 235

SOCIETY NOTES—

Proceedings Richmond Academy of Medicine and Surgery . . . . . 237

Transactions of the British Medical Association . . . . . 239

CURRENT MEDICAL LITERATURE—

Pseudo-Chancre . . . . . 244

ABSTRACTS AND SELECTIONS—

Order Concerning the Importation of Cattle from the Republic of Mexico . . . . . 245

National Conference of State Boards of Health . . . . . 246

EDITORIAL—

A Fishy Novel, by a Texas Doctor . . . . . 247

A Texas Home for Epileptics . . . . . 251

Vanity of Vanities . . . . . 254

The Doctor and Politics . . . . . 255

The Key-Note to Success . . . . . 256

PROGRESS IN MEDICINE—

The Treatment of Intermittent Fever . . . . . 257

MEDICAL NEWS AND MISCELLANY—

Many Interesting Items . . . . . 260

BOOK NOTICES— . . . . . 279

PUBLISHERS' NOTES . . . . . 280

